# Two Nimrod receptors, NimC1 and Eater, synergistically contribute to bacterial phagocytosis in *Drosophila melanogaster*

**DOI:** 10.1111/febs.14857

**Published:** 2019-05-13

**Authors:** Claudia Melcarne, Elodie Ramond, Jan Dudzic, Andrew J. Bretscher, Éva Kurucz, István Andó, Bruno Lemaitre

**Affiliations:** ^1^ Global Health Institute School of Life Sciences École Polytechnique Fédérale de Lausanne (EPFL) Switzerland; ^2^ Institute of Genetics Biological Research Centre of the Hungarian Academy of Sciences Szeged Hungary

**Keywords:** *Drosophila*, haemocytes, innate immunity, Nimrod, phagocytosis

## Abstract

Eater and NimC1 are transmembrane receptors of the *Drosophila* Nimrod family, specifically expressed in haemocytes, the insect blood cells. Previous *ex vivo* and *in vivo* RNAi studies have pointed to their role in the phagocytosis of bacteria. Here, we have created a novel *NimC1* null mutant to re‐evaluate the role of NimC1, alone or in combination with Eater, in the cellular immune response. We show that NimC1 functions as an adhesion molecule *ex vivo*, but in contrast to Eater it is not required for haemocyte sessility *in vivo*. *Ex vivo* phagocytosis assays and electron microscopy experiments confirmed that Eater is the main phagocytic receptor for Gram‐positive, but not Gram‐negative bacteria, and contributes to microbe tethering to haemocytes. Surprisingly, *NimC1* deletion did not impair phagocytosis of bacteria, nor their adhesion to the haemocytes. However, phagocytosis of both types of bacteria was almost abolished in *NimC1* ^*1*^ *;eater* ^*1*^ haemocytes. This indicates that both receptors contribute synergistically to the phagocytosis of bacteria, but that Eater can bypass the requirement for NimC1. Finally, we uncovered that NimC1, but not Eater, is essential for uptake of latex beads and zymosan particles. We conclude that Eater and NimC1 are the two main receptors for phagocytosis of bacteria in *Drosophila,* and that each receptor likely plays distinct roles in microbial uptake.

## Graphical abstract

Eater and NimC1 are transmembrane receptors belonging to the *Drosophila* Nimrod family. These receptors are expressed in insect blood cells (haemocytes) and have been implicated in bacterial phagocytosis. In this study, Bruno Lemaitre and colleagues created a novel *NimC1* null mutant and used this to characterize the role of NimC1 in cellular immune responses, alone and in combination with Eater. The authors reveal that Eater and NimC1 contribute synergistically to the initial step of phagocytosis, notably adhesion to bacteria, and that the receptors play distinct roles in this process.

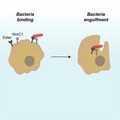

**Abbreviations**
DAPI: 4′,6‐ diamidino‐2‐phenylindole
EdU: 5‐ethynyl‐2′‐deoxyuridine
Hml: Hemolectin
Nim: Nimrod
PTU: phenylthiourea
SEM: scanning electron microscopy
TEM: transmission electron microscopy

## Introduction

Phagocytosis is an ancient and evolutionarily conserved process, generally defined as the cellular uptake of particles bigger than 0.5 μm. Phagocytosis is an important feeding mechanism in primitive and unicellular organisms, such as amoeba 1. In higher organisms, phagocytosis is performed by dedicated cells (phagocytes) and is used as a powerful process to internalize and eliminate pathogens, as well as to trigger host inflammation 2. Moreover, phagocytosis contributes to tissue homeostasis and embryonic development, mainly via the removal of apoptotic corpses 3. Phagocytosis is a complex membrane‐driven process guided by the actin cytoskeleton of the host phagocytic cell. It involves the recognition and subsequent binding of the microbe by surface receptors. These interactions are essential to activate intracellular signalling pathways that finally culminate in the formation of the phagosome 4. Several studies have highlighted similarities between the phagocytic machinery of *Drosophila* and mammals, such as the involvement of actin and actin‐related proteins 5, 6, 7. *Drosophila melanogaster* harbours highly efficient phagocytes, called plasmatocytes, which originate from multipotent progenitors (prohaemocytes). In healthy larvae, prohaemocytes can differentiate into two mature haemocyte types: plasmatocytes and crystal cells. While the later are involved in the melanization response 8, plasmatocytes are professional phagocytes sharing functional features with mammalian macrophages, and represent the most abundant haemocyte class at all developmental stages. They play a key role in bacterial clearance during infection, as well as in the removal of apoptotic corpses 9, 10. The ability of *Drosophila* haemocytes to perform efficient phagocytosis relies on the expression of specific cell surface receptors that can bind particles and induce their engulfment. While many receptors have been implicated in bacterial phagocytosis, their specific involvement or individual contribution is less clear 11, 12. In this paper, we have characterized the phagocytic role of NimC1 and Eater, two EGF‐like repeat Nimrod surface receptors specifically expressed in haemocytes 13, 14. The Nimrod family of proteins is characterized by the presence of epidermal growth factor (EGF)‐like domains, also called ‘NIM repeats’. This family comprises a cluster of 10 genes (NimA, NimB1‐5 and NimC1‐4) encoded by genes clustered on the chromosome II, and two related haemocyte surface receptors, Eater and Draper, encoded by genes on chromosome 3 14, 15. Early studies have shown the implication of some Nimrod C‐type proteins in bacterial phagocytosis (Eater and NimC1) 13, 14, 16 or engulfment of apoptotic bodies (Draper and NimC4/SIMU) 17, 18. More recently, the Eater transmembrane receptor has also been involved in haemocyte adhesion and sessility 16. Nimrod C1 (NimC1) is a 90‐kDa transmembrane protein characterized by 10 NIM repeats in its extracellular region, a single transmembrane domain and a short cytosolic tail with unknown function 14. NimC1 has been initially identified as the antigen of a haemocyte‐specific antibody (P1), being involved in phagocytosis of bacteria 14. Kurucz *et al*. 14 showed that *NimC1* silencing by RNAi decreases *Staphylococcus aureus* uptake by plasmatocytes, whereas its overexpression in S2 cells enhances phagocytosis of both *S. aureus* and *Escherichia coli* bacteria and makes the cells highly adherent. Here, we generated a null mutation in *NimC1* by homologous recombination (called *NimC1* ^*1*^) and revisited its function in haemocyte‐mediated immunity. Moreover, we recombined the *NimC1* mutation with the previously described *eater* ^*1*^ mutant 16, generating a *NimC1* ^*1*^ *;eater* ^*1*^ double mutant. Using these genetic tools, we first show the involvement of NimC1 in *ex vivo* cell adhesion and in the regulation of haemocyte proliferation. Contrasting with previous RNAi studies 14, our *ex vivo* phagocytosis assays demonstrate that NimC1 is not required for phagocytosis of Gram‐positive or Gram‐negative bacteria. Nevertheless, we show that this Nimrod receptor contributes to the uptake of latex beads and zymosan yeast particles. The use of the *NimC1* ^*1*^ *;eater* ^*1*^ double mutant not only reconfirmed Eater as the main Gram‐positive engulfing receptor, but, more importantly, revealed a synergistic action of NimC1 and Eater in microbe phagocytosis. *NimC1* ^*1*^ *;eater* ^*1*^ haemocytes from third instar larvae, failed indeed to phagocytose any type of bacteria. Collectively, our study points to a major role of NimC1 and Eater in the phagocytosis of bacteria, and suggests that those proteins likely play distinct roles in microbial uptake, as tethering and docking receptors.

## Results

### Generation of a *NimC1* null mutant by homologous recombination

**Figure 1:**
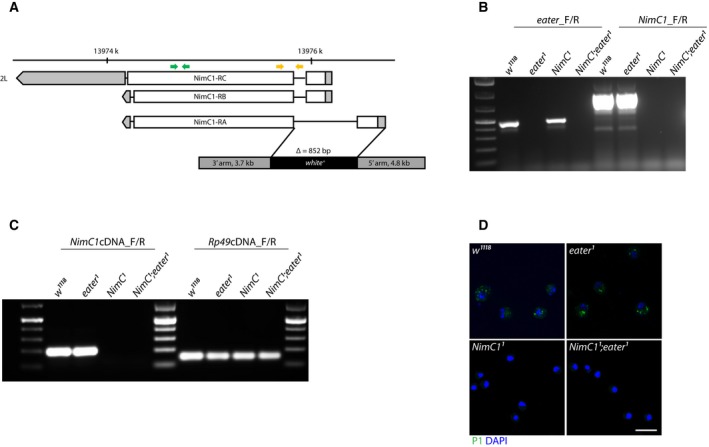
Gene targeting and deletion of *NimC1*. (A) *NimC1* gene deletion by homologous recombination. The *NimC1* gene is located on the left (L) arm of chromosome 2 and it encodes three isoforms. White and grey boxes represent exons and UTR regions respectively. Eye colour was transformed from white to red by the *white* ^+^ marker. Yellow and green arrows represent, respectively, the location of primers used in (B) and (C). (B) PCR genotyping confirming the targeted deletion of the *NimC1* gene, whereas the *eater* locus was not affected. (C) RT‐PCRs confirming functional deletion of *NimC1*. (D) NimC1 (P1) staining (green) of third instar larval haemocytes from the indicated genotypes. Cell nuclei are shown in DAPI (blue). The immunostaining was performed as previously described in 59. Scale bar: 20 μm.

In order to characterize *NimC1* functions, we generated a null mutant by deleting the corresponding *NimC1* gene region. The deletion removes the ATG translation start site and the following 852‐bp sequence. The knockout was performed in the *w* ^*1118*^ background, using homologous recombination 19, which also leads to the insertion of a 7.9‐kb cassette carrying the *white* ^*+*^ gene (Fig. 1A,B). Functional deletion of *NimC1* was confirmed by RT‐PCR performed on total RNA and by P1 (anti‐NimC1 antibody 14) immunostaining (Fig. 1C,D). As *NimC*1 is specifically expressed in haemocytes and has been implicated in phagocytosis, we combined the *NimC1* mutation with the previously described *eater* ^*1*^ null mutant 16, generating a double mutant *NimC1* ^*1*^ *;eater* ^*1*^ (Fig. 1). Both *NimC1* ^*1*^ and *NimC1* ^*1*^ *;eater* ^*1*^ flies were viable and did not show any developmental defect. For overexpression studies, we also generated flies containing the *NimC1* gene downstream of the *UAS* promoter. Using these tools, we characterized the function of NimC1 focussing on haemocytes of third instar larvae.

### *NimC1*‐deficient haemocytes show adhesion defects *in vitro*

**Figure 2:**
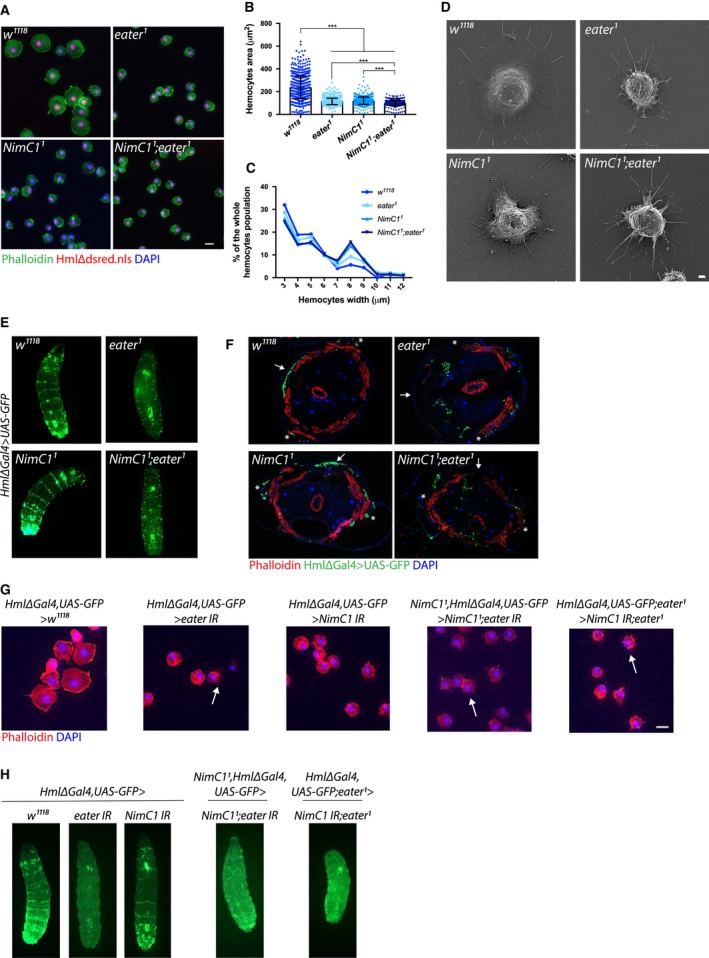
*NimC1* ^*1*^ haemocytes show spreading defects *in vitro*. (A) Representative images of fixed haemocytes from *w* ^*1118*^, *eater* ^1^, *NimC1* ^1^ and *NimC1* ^*1*^ *;eater* ^*1*^ L3 wandering larvae combined with *HmlΔdsred.nls* marker (red). Haemocytes of the indicated genotypes were extracted by larval bleeding, allowed to spread for 30 min on a glass slide, and stained with AlexaFluor™488 phalloidin (green). Scale bar: 10 μm. (B) Mean cell area quantification of fixed *HmlΔdsred.nls* haemocytes spread for 30 min on slides and stained with AlexaFluor488 phalloidin. Cell area of 750 cells was quantified using the cellprofiler software (www.cellprofiler.org). (C) Size distribution of free‐floating haemocytes from *w* ^*1118*^, *eater* ^1^, *NimC1* ^1^ and *NimC1* ^*1*^ *;eater* ^*1*^ L3 wandering larvae. Haemocyte size was measured with TALI imaged‐based cytometer directly after larval bleeding of more than 7000 cells per genotype. (D) Representative SEM images of spread haemocytes from the indicated genotypes of L3 wandering larvae. Scale bar: 1 μm. (E) Whole larva images of *w* ^*1118*^, *eater* ^*1*^, *NimC1* ^*1*^ and *NimC1* ^*1*^ *;eater* ^*1*^ third instar larvae specifically expressing *UAS‐GFP* in plasmatocytes driven by *Hml∆‐GAL4*. The dorsal side of the animal is shown. (F) Cross sections of the indicated genotypes from L3 wandering larvae combined with *HmlΔGal4>UAS‐GFP* (green). Rhodamine phalloidin staining (red) was performed after larva cross sectioning. Cell nuclei are shown in DAPI (blue). White arrows and asterisk indicate dorsal and lateral side of the animal respectively. (G) Representative images of fixed haemocytes from the indicated genotypes of L3 wandering larvae, stained with rhodamine phalloidin (red). Cell nuclei were stained with DAPI (blue). Arrows indicate the presence of filopodia in the corresponding genotype, found also in the *eater* deletion mutants. Scale bar: 10 μm. (H) Whole larva images of third instar larvae of the indicated genotypes, specifically expressing GFP in plasmatocytes driven by *Hml∆‐GAL4*. The dorsal side of the animal is shown. ***P <* *0.001 by Mann–Whitney tests.

Eater has been involved in haemocyte adhesion and sessility 16. Given the structural similarities between NimC1 and Eater 14, we first investigated the role of NimC1 in cell adhesion. We observed that the cell area of *NimC1* ^*1*^ *‐* and *eater* ^*1*^ *‐*adherent haemocytes was decreased compared to that of *w* ^*1118*^ wild‐type control (Fig. 2A) 16. Notably, the cell area of *NimC1* ^*1*^ *;eater* ^*1*^‐adherent haemocytes was significantly smaller than that of single mutants. Quantification analysis revealed that wild‐type haemocytes have a mean cell area of 237 μm^2^, while *NimC1* ^*1*^ *, eater* ^*1*^ and *NimC1* ^*1*^ *;eater* ^*1*^ mutants have 120, 114 and 99.7 μm^2^ respectively (Fig. 2B). Image‐based cytometry analysis of free‐floating haemocytes revealed that the spreading defects observed in our mutants were not due to an inherently smaller cell size (Fig. 2C). In order to get a deeper insight into these adhesion defects, we investigated haemocyte morphology by scanning electron microscopy (SEM). Lamellipodia are a key feature of highly motile cells, playing a central role in cell movement and migration 20. They represent flat cellular protrusion, characterized by an enriched network of branched actin filaments. Filopodia, instead, are rather used by the cell to sense the surrounding microenvironment, and consist of parallel actin filaments that emerge from the lamellipodium. Spread plasmatocytes from wild‐type larvae appeared as round adherent cells with a central bulge within the cell body, from which lamellipodia and filopodia extended (Fig. 2D). *NimC1* and *eater* null haemocytes were still able to form narrow filopodia projections. However, both single and double mutants showed an obvious lamellipodium decreased region compared to wild‐type control (Fig. 2D). Collectively, our results point to a role of NimC1 in haemocyte spreading and lamellipodia extension.

In the *Drosophila* larva, circulating haemocytes can attach to the inner layer of the cuticle, forming striped patterns along the dorsal vessel, and lateral patches in association with the endings of peripheral neurons 8, 21, 22, 23. These subepidermal sessile compartments are known as haematopoietic pockets 21, 23, 24, 25, 26. Previous work has shown that *eater* larvae lack the sessile haemocyte compartment and have all peripheral haemocytes in circulation 16. To further investigate whether the *NimC1* deletion affects sessility, we explored haemocyte localization using the haemocyte marker *Hml∆Gal4,UAS>GFP* by whole larva imaging and cross‐section visualization. In *NimC1* ^*1*^ *,Hml∆Gal4,UAS‐GFP* third instar (L3) wandering larvae, haemocytes were still able to enter the sessile state, forming dorsal and lateral patches (Fig. 2E,F). In contrast, both *eater* ^*1*^ and *NimC1* ^*1*^ *;eater* ^*1*^ larvae lacked sessile haemocytes, all plasmatocytes being in circulation (Fig. 2E,F). *In vivo* RNAi targeting *NimC1* confirmed the haemocyte adhesion defect observed with the null mutant (Fig. 2G,H). This indicates that the observed phenotypes were indeed caused by the deletion of *NimC1* and not the genetic background. Altogether, our data indicate that NimC1 contributes to haemocyte adhesion *ex vivo*, but in contrast to Eater, it is not directly required for haemocyte sessility *in vivo*.

### *NimC1* null larvae have an increased number of haemocytes

**Figure 3:**
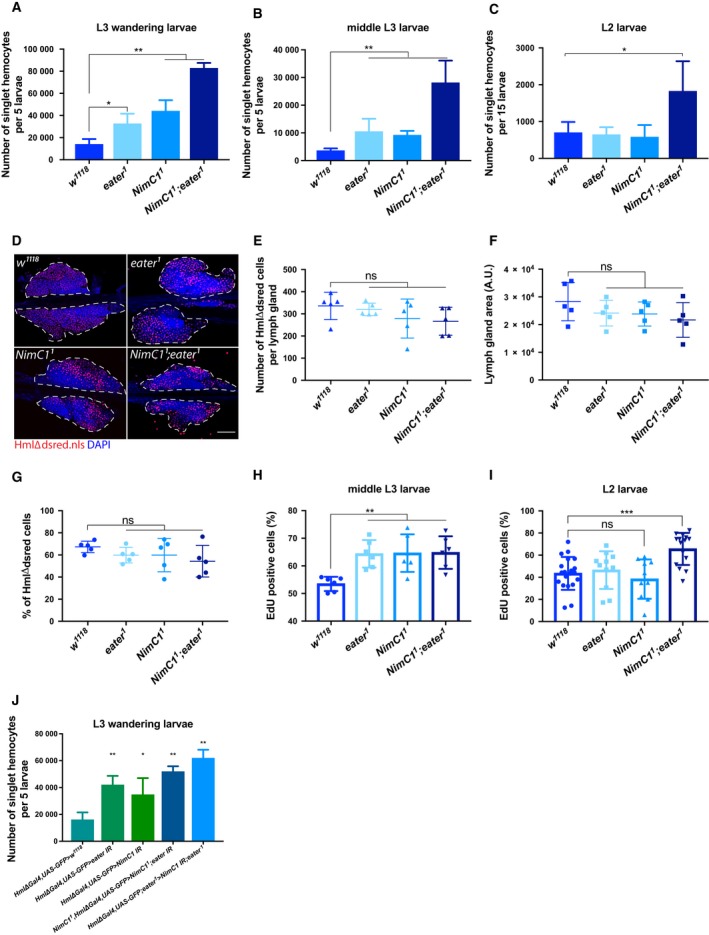
*NimC1* ^*1*^ and *NimC1* ^*1*^ *;eater* ^*1*^ larvae give rise to a higher number of haemocytes. Number of singlet peripheral haemocytes per 5 L3 wandering (A), 5 middle L3 (B) and 15 L2 (C) larvae of *w* ^*1118*^, *eater* ^1^, *NimC1* ^1^, *NimC1* ^*1*^ *;eater* ^*1*^ combined with *HmlΔdsred.nls*. In (A–C) data are represented as mean ± SD from five independent experiments. (D) Representative confocal images of dissected lymph glands from *w* ^*1118*^, *eater* ^1^, *NimC1* ^1^, *NimC1* ^*1*^ *;eater* ^*1*^ third instar larvae combined with *HmlΔdsred.nls* haemocyte marker (red). Lymph gland primary lobes are shown and boundaries are delimited by a white dashed line. Cell nuclei were stained with DAPI (blue) after paraformaldehyde fixation. Scale bar: 50 μm. Images were acquired with Zeiss LSM700 confocal microscope. (E) Absolute number of *HmlΔdsred.nls* cells in primary lymph gland lobes of the indicated genotypes. (F) Lymph gland area quantification in the indicated genotypes, performed by using imagej software tool (www.imagej.nih.gov). A.U., arbitrary units. (G) Percentage of *HmlΔdsred.nls* cells upon DAPI‐positive cells in primary lymph gland lobes of the indicated genotypes. Five primary lymph gland primary lobes per genotypes were analysed in (E–G). Percentage of EdU‐positive cells upon *HmlΔdsred.nls* cells in middle L3 (H) and L2 larval (I) stage of *w* ^*1118*^, *eater* ^1^, *NimC1* ^1^, *NimC1* ^*1*^ *;eater* ^*1*^. A number of at least six animals was used for each genotype. (J) Number of singlet peripheral haemocytes per five L3 wandering larvae of the indicated genotypes. Data are represented as mean ± SD from five independent experiments. *P <* *0.05, **P <* *0.01, ***P <* *0.001 by Mann–Whitney test.

*Drosophila* haematopoiesis occurs in two successive waves. A first set of haemocytes is produced during embryogenesis, giving rise to a defined number of plasmatocytes and crystal cells. This embryonic haemocyte population expands in number during the following larval stages. The second haemocyte lineage derives from the lymph gland, a specialized organ that develops along all larval stages. The lymph gland acts as a reservoir of both prohaemocytes and mature haemocytes, which are released at the onset of metamorphosis or upon parasitization 8, 27, 28, 29, 30. Finally, accumulating evidence suggests that the sessile haematopoietic pockets also function as an active peripheral haematopoietic niche 21, 23, 26. In order to further investigate the role of NimC1, and its potential interaction with Eater in peripheral haematopoiesis, we counted by flow cytometry the number of all the peripheral haemocyte populations (i.e. both sessile and circulating). Larvae containing the haemocyte marker *Hml∆dsred.nls*, combined with the *NimC1* and *eater* null mutants, were used (Fig. 3A–C). Our study confirmed that *eater* L3 wandering mutant larvae have more haemocytes than the wild‐type 16 (Fig. 3A). Similarly, *NimC1* ^*1*^ third instar larvae have 3.2 times more circulating haemocytes compared to the wild‐type (Fig. 3A). As *NimC1* ^*1*^ L2 larvae have a wild‐type like number of haemocytes, the increase in haemocyte counts in this mutant takes place at the end of larval development (Fig. 3C). Surprisingly, haemocyte number was six times higher in *NimC1* ^*1*^ *;eater* ^*1*^ double mutant L3 larvae (Fig. 3A), suggesting that *eater* and *NimC1* additively regulate haemocyte counts. A higher haemocyte number was already observed in second instar larvae in the double mutant (Fig. 3C). We next investigated whether lymph glands from third instar mutant larvae had an increased number of mature haemocytes compared to wild‐type. Visual count of *Hml∆dsred.nls*‐positive cells from fixed primary lymph gland lobes revealed no major differences between mutants and wild‐type, although a decreased trend in haemocyte number in single and double mutants could be observed (Fig. 3D,E). In agreement with this observation, primary lymph gland lobes of *eater* and *NimC1* mutants showed a modest reduced area compared to control, which was not statistically significant (Fig. 3F). Nevertheless, the ratio of *Hml∆dsred.nls*‐positive cells to the all primary lymph gland cell population (i.e. DAPI positive), was not significantly altered between wild‐type and mutants (Fig. 3G).

**Figure 4:**
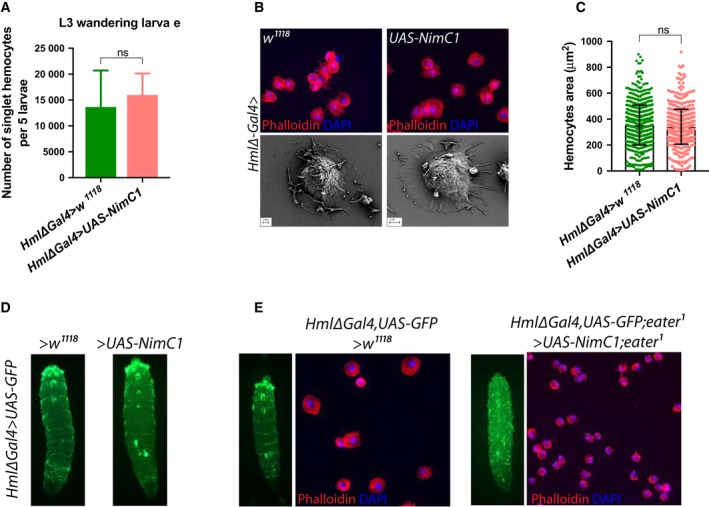
*NimC1* overexpression does not alter haemocyte number and adhesive properties. (A) Number of singlet peripheral haemocytes in third instar wandering larvae is not affected upon NimC1 overexpression. Results are represented as a sum of five animals with the indicated genotypes. Data are represented as mean ± SD from five independent experiments. (B) Upper panel: Representative images for fixed haemocytes from *HmlΔGal4*>*w* ^*1118*^ and *HmlΔGal4*>*UAS‐NimC1* L3 wandering larvae stained with rhodamine phalloidin (red). Cell nuclei are shown in DAPI (blue). Bottom panel: scanning electron micrographs on spread haemocytes from *HmlΔGal4*>*w* ^*1118*^ and *HmlΔGal4*>*UAS‐NimC1* of L3 wandering larvae. (C) Mean cell area quantification of fixed haemocytes of the indicated genotypes, spread for 30 min on slides and stained with AlexaFluor488 phalloidin. Cell area of 750 cells was quantified using the cellprofiler software. (D) Whole larva imaging of *HmlΔGal4,UAS‐GFP>w* ^*1118*^ and *HmlΔGal4,UAS‐GFP>UAS‐NimC1* shows no major difference in haemocyte localization pattern and adherence when NimC1 is specifically overexpressed in haemocytes. The dorsal side of the animal is shown. (E) Whole larva imaging and spreading assay showing the absence of rescue when overexpressing *NimC1* in an *eater* mutant background. Data in (A) and (C) were analysed by Mann–Whitney test. ns, not significant.

We then decided to explore whether the increase in peripheral haemocytes count observed in our mutants (Fig. 3A–C) was caused by a higher proliferation rate. EdU incorporation experiments revealed that *NimC1* ^*1*^ and *eater* ^*1*^ single mutants have a higher frequency of peripheral proliferating haemocytes compared to wild‐type in middle L3 but not L2 larvae (Fig. 3H,I). The higher proliferation rates might, therefore, explain the increased number of haemocyte counts in both L3 wandering (Fig. 3A) and middle L3 (Fig. 3B) larvae. Interestingly, we found that both haemocyte count and mitotic rate were higher in *NimC1* ^*1*^ *;eater* ^*1*^ in L2 and L3 larvae indicating that both receptors additively regulate haemocyte proliferation levels (Fig. 3A–C,H,I). The higher haemocyte count in *NimC1* mutant larvae was phenocopied when using an *in vivo* RNAi approach to silence *NimC1* (Fig. 3J). Of note, overexpression of *NimC1*, using the *Hml∆‐Gal4* plasmatocyte driver, did not increase the peripheral haemocyte count (Fig. 4A), nor their adhesion properties (Fig. 4B–D). Overexpression of *NimC1* in haemocytes from *eater*‐deficient larvae did not rescue the lack of sessility phenotype and the *ex vivo* adhesion defect caused by the absence of *eater* (Fig. 4E).

We also investigated a possible role of NimC1 in crystal cell and lamellocyte differentiation.

**Figure 5:**
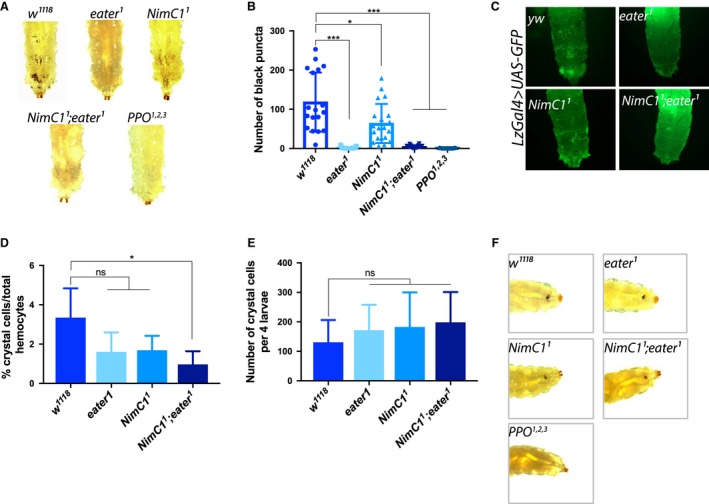
Sessile and circulating crystal cell populations are mildly affected in *NimC1* ^*1*^ larvae. (A) Heating larvae induces the spontaneous activation of the prophenoloxidase zymogen within crystal cells, leading to their blackening 60. Consequently, the population of crystal cells attached under the cuticle becomes visible as black puncta. Sessile crystal cell numbers were mildly reduced in *NimC1* ^*1*^ larvae, while being almost completely absent in *eater* ^*1*^ and *NimC1* ^*1*^ *;eater* ^*1*^ larvae. Shown are representative images of *w* ^*1118*^, *eater* ^*1*^, *NimC1* ^*1*^ and *NimC1* ^*1*^ *;eater* ^*1*^ **L3 wandering larvae after heat treatment at 67 °C for 20 min. (B) Black puncta count from the three posterior‐most segments of heated *w* ^*1118*^ *, eater* ^*1*^ *, NimC1* ^*1*^ *and NimC1* ^*1*^ *;eater* ^*1*^ third instar larvae. A number of at least 18 animals was used for each genotype. (C) *In vivo* imaging using the *lzGal4>UAS‐GFP* crystal cell marker confirmed the previous observations. Shown is the dorsal view of the five posterior‐most segments in *yw*,* eater* ^*1*^, *NimC1* ^*1*^ and *NimC1* ^*1*^ *;eater* ^*1 *^L3 wandering larvae, previously combined with the crystal cell lineage marker *lzGal4>UAS‐GFP*. (D‐E) Flow cytometry counting of *lzGal4>UAS‐GFP*–positive cells revealed a wild‐type number of crystal cells in *NimC1* and *eater*‐deficient L3 wandering larvae (D), and a moderately decreased ratio of crystal cells over the total haemocyte population (E), pointing to a mild defect in sessility but not in the general ability to differentiate crystal cells. Data are represented as mean ± SD from five independent experiments. (F) Melanization response to epithelial wounding is wild‐type like in *eater* and *NimC1* mutants larvae. Representative images of melanized larvae were acquired 20 min after pricking. In (A) and (F) *PPO* ^*1,2,3*^ mutant larvae were used as negative control. *P <* *0.05, ***P <* *0.001, by Mann–Whitney tests. ns: not significant.

**Figure 6:**
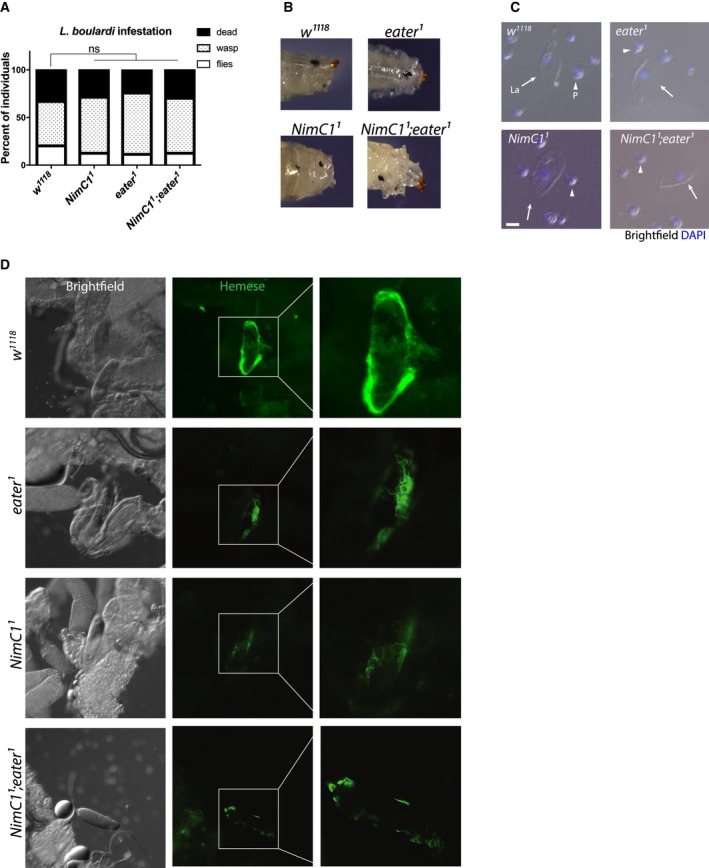
*NimC1* mutants do not show any major encapsulation defects after wasp infestation. (A) Quantification of emerging *Drosophila melanogaster* adult, *Leptopilina boulardi* wasp, and dead animals following parasitization with the parasitoid wasp *L. boulardi*. Data are shown as a sum of three experiments, with a total of 90 animals for each genotype. Data were analysed using Chi‐square statistical test (*P*‐value > 0.05). ns, not significant. (B) Shown are representative images of melanized wasp eggs in *w* ^*1118*^ control and mutants, 70 h after *L. boulardi* infestation. (C) Lamellocyte differentiation is observed in *eater* and *NimC1* mutants upon wasp infestation. Representative images showing circulating haemocytes 70 h after *L. boulardi* infestation. Arrows and arrowheads indicate haemocytes with lamellocyte (La) and plasmatocyte (P) morphology respectively. Cell nuclei were stained with DAPI (blue) Scale bar: 20 μm. (D) Representative images showing early wasp egg recognition by peripheral plasmatocytes (haemocytes stained with anti‐Hemese antibody 61, green) 20 h after infestation. Haemocytes of both wild‐type and *eater* and *NimC1* mutants attached to the eggs. However, single and double‐mutant plasmatocytes adhere with slightly decreased spreading ability compared to the wild‐type.

**Figure 7:**
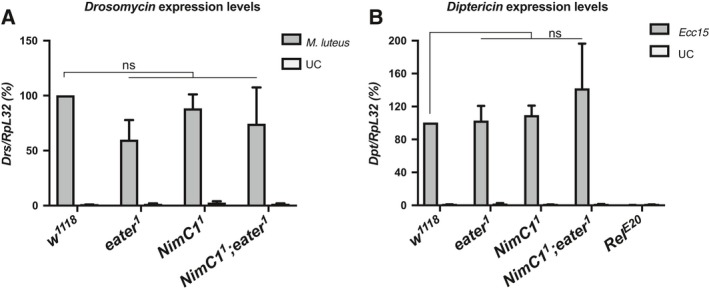
NimC1 is not involved in humoral immunity. Expression levels of *Diptericin* (A) and *Drosomycin* (B) relative to *RpL32* in *w* ^*1118*^, *eater* ^1^, *NimC1* ^1^ and *NimC1* ^*1*^ *;eater* ^*1*^ third instar larvae. Total RNA from infected animals was extracted 4 h after *Ecc15* or *Micrococcus luteus* septic injury. UC, unchallenged controls. The Imd pathway mutant *Relish* (*Rel* ^*E20*^) was used as an immune‐deficient control in (A). Data are represented as mean ± SD from 3 independent experiments ns: not significant, by Mann–Whitney test.

Crystal cells are the second haemocyte type present in noninfected larvae, specifically involved in the melanization response and wound healing 31. Crystal cells can be found in both the sessile and circulating state. Recent studies have shown that a fraction of those cells derive from sessile plasmatocyte by transdifferentiation 26. Consequently, crystal cells need sessile plasmatocytes to be, themselves, sessile 16. Lamellocytes are barely present in healthy larvae, but can differentiate from plasmatocytes 25, 32 or prohaemocytes 33 in response to specific stress signals, such as parasitization. They are thought to play an essential role in encapsulation of parasitoid wasp eggs. Our study indicates that *NimC1* mutants retain the ability to differentiate fully mature crystal cells (Fig. 5). Moreover, our data also show that the *NimC1* deletion does not affect the ability to encapsulate parasitoid wasp eggs (Fig. 6). Finally, we did not uncover any role of NimC1 in the systemic antimicrobial response of larvae against Gram‐positive (*Micrococcus luteus*) or Gram‐negative bacteria (*Erwinia carotovora carotovora*), as revealed by the wild‐type–like induction of *Diptericin* and *Drosomycin* gene expression, two target genes of the Imd and Toll pathways respectively 34 (Fig. 7).

### NimC1 contributes with Eater to phagocytosis of bacteria

**Figure 8:**
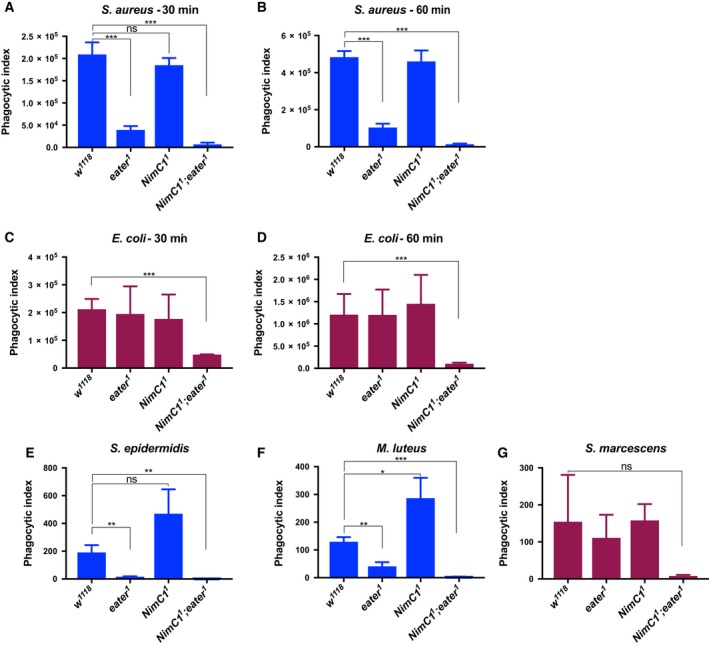
NimC1 contributes with Eater to phagocytosis of Gram‐positive and Gram‐negative bacteria. *Ex vivo* phagocytosis assay using *Staphylococcus aureus* (A, B) and *Escherichia coli* (C, D) AlexaFluor™488 BioParticles™ (Invitrogen). *HmlΔdsred.nls* haemocytes from third instar wandering larvae were incubated with the particles for 60 (A, C) or 30 (B, D) min at room temperature. In (A–D) data are represented as mean ± SD from four independent experiments. *Ex vivo* phagocytosis assay using the Gram‐positive *Staphylococcus epidermidis* (E), *Micrococcus luteus* (F) and Gram‐negative *Serratia marcescens* (G) bacteria. Bacteria were first heat inactivated and subsequently labelled with fluorescein isothiocynate (FITC). Haemocytes from third instar wandering larvae of the indicated genotypes were incubated with the bacteria for 40 min at room temperature. In (E, F) data are represented as mean ± SD from three independent experiments. In (A–G), phagocytosis was quantified by flow cytometry, and the fluorescence of extracellular particles quenched by adding trypan blue. *P <* *0.05, **P <* *0.01, ***P <* *0.001, by Student *t* tests. ns: not significant.

A previous *in vivo* RNAi approach had revealed a role of NimC1 in the phagocytosis of Gram‐positive bacteria 14. We used the *NimC1* deletion to further elucidate the requirement of this receptor in bacterial uptake by performing *ex vivo* phagocytosis assays at two different time points (early‐30 min and late‐60 min). As previously reported 16, *eater* null mutant haemocytes were impaired in their capacity to phagocytose the Gram‐positive bacterium *S. aureus* (Fig. 8A,B), but not the Gram‐negative bacterium *E. coli* (Fig. 8C,D). In contrast to the previous RNAi experiments 14, loss of *NimC1* affected neither the phagocytosis of *S. aureus* nor that of *E. coli* (Fig. 8A–D). However, haemocytes derived from *NimC1;eater* mutant larvae were not only severely impaired in the phagocytosis of *S. aureus* (Fig. 8A,B), as expected, but also of *E. coli* (Fig. 8C,D). This indicates that Eater and NimC1 contribute redundantly to the phagocytosis of Gram‐negative bacteria, as the presence of Eater or NimC1 is able to compensate for the absence of the other. The use of a double mutant also revealed a contribution of NimC1 to the phagocytosis of *S. aureus*, although Eater plays the predominant role. To further confirm these phagocytosis defects, we extended the analysis to two additional Gram‐positive (*Staphylococcus epidermidis*,* M. luteus*) (Fig. 8E,F) and one Gram‐negative (*Serratia marcescens*) (Fig. 8G) bacteria. Phagocytosis of all those microbes was not impaired in *NimC1* ^*1*^ null haemocytes. However, *NimC1* ^*1*^ *;eater* ^*1*^ double mutant haemocytes showed a strongly reduced phagocytosis for both the Gram‐positive bacteria *S. epidermidis* and *M. luteus* (Fig. 8E,F), and the Gram‐negative bacterium *S. marcescens* (although statistically nonsignificant due to the high variability of the wild‐type) (Fig. 8G). Those data further confirmed our initial findings (Fig. 8A–D). Interestingly, *NimC1* null haemocytes showed a higher phagocytic index, when compared to wild‐type, for *S. epidermidis* and *M. luteus* bacteria. We hypothesized that the absence of NimC1 could trigger a compensatory pathway in plasmatocytes, specific for certain bacteria, in order to fulfil NimC1 phagocytic functions. The signalling of this putative compensatory pathway, that would eventually finally lead to a higher bacteria uptake by plasmatocytes, might be dependent on Eater, given the dramatically reduced phagocytic ability of the double mutant.

### Eater and NimC1 receptors play a critical role in adhesion to bacteria

**Figure 9:**
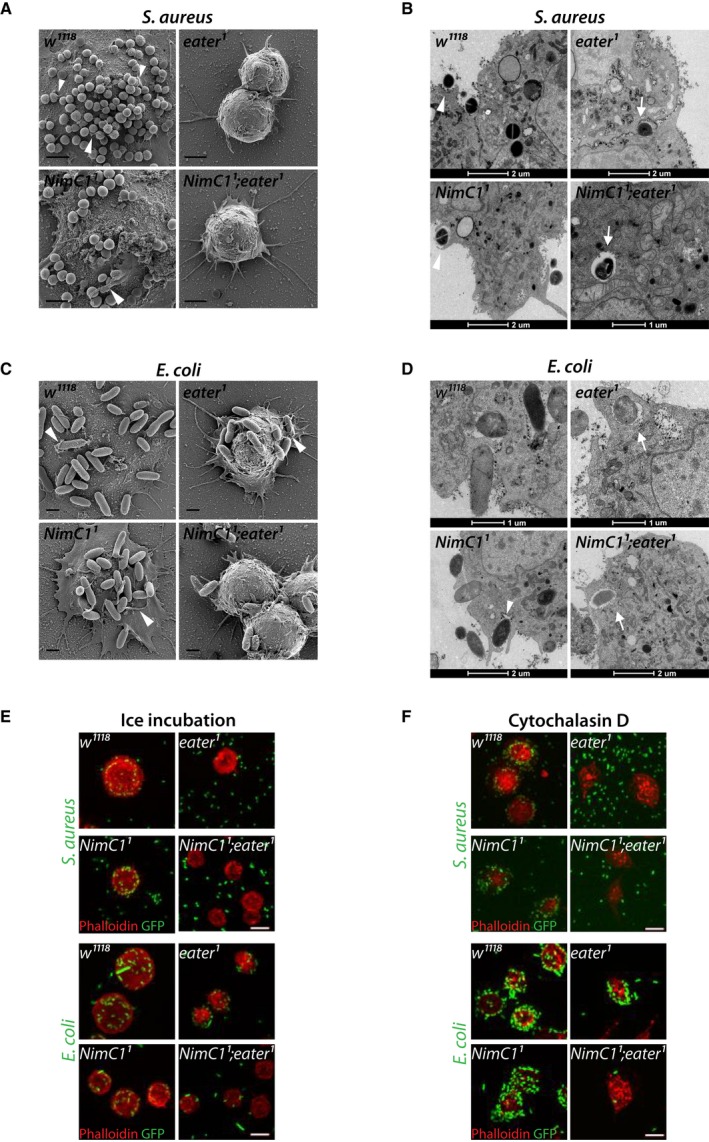
*eater* ^*1*^ and *NimC1* ^*1*^ *;eater* ^*1*^ haemocytes show bacteria adhesion defects. (A) Representative SEM images of haemocytes from the indicated genotypes of L3 wandering larvae after 30 min incubation with *Staphylococcus aureus* live bacteria, at room temperature. Scale bar: 2 μm. (B) Representative transmission electron micrographs of haemocytes from the indicated genotypes of L3 wandering larvae after 60 min incubation with *S. aureus* live bacteria, at room temperature. (C) Representative SEM images of haemocytes from the indicated genotypes of L3 wandering larvae after 30 min incubation with *Escherichia coli* live bacteria, at room temperature. Scale bar: 1 μm. (D) Representative transmission electron micrographs of haemocytes from the indicated genotypes of L3 wandering larvae after 60 min incubation with *E. coli* live bacteria, at room temperature. Arrowheads and arrows indicate haemocyte membrane protrusions and internalized bacteria respectively. (E, F) Haemocytes from the corresponding genotypes were incubated with *S. aureus* GFP (green) or *E. coli* GFP (green) live bacteria on ice (E), or with Cytochalasin D (F), for 1 h (see Material and Methods section for further details). After fixation with 4% paraformaldehyde, haemocytes were stained with rhodamine phalloidin (red). Scale bar: 10 μm.

To better understand the cause of *eater* ^*1*^ and *NimC1* ^*1*^;*eater* ^*1*^ phagocytosis defects, and thereby to elucidate the unique role of these receptors in bacterial uptake, we performed scanning and transmission (TEM) electron microscopy experiments. Both these techniques allow following the different membrane‐driven events during the phagocytosis process. Haemocytes from the corresponding genotypes were incubated with either *E. coli* or *S. aureus* live bacteria for 30 min to evaluate bacterial adhesion by SEM, and to follow bacterial uptake at 60 min by TEM. In wild‐type and *NimC1* ^*1*^ haemocytes incubated with *S. aureus*, we observed plasma membrane remodelling, with the formation of a phagocytic cup and pseudopod protrusions that progressively surrounded bacteria, finally leading to their engulfment (Fig. 9A,B white arrowheads). Similar observations were made for *wild‐type*,* NimC1* ^*1*^ and *eater* ^*1*^ haemocytes incubated with *E. coli* bacteria (Fig. 9C,D). Surprisingly, upon incubation of *eater* ^*1*^ and *NimC1* ^*1*^ *;eater* ^*1*^ haemocytes with *S. aureus*, no bacteria were present on the cell surface (Fig. 9A). A decreased level of bacteria adherence was also observed in *NimC1* ^*1*^ *;eater* ^*1*^ haemocytes incubated with *E. coli* (Fig. 9C). In accordance with SEM experiments, transmitted electron micrographs showed numerous engulfment events in *wild‐type* and *NimC1* ^*1*^ haemocytes with *S. aureus* bacteria (Fig. 9B, arrows), as well as for *E. coli* in *wild‐type*,* NimC1* ^*1*^ and *eater* ^*1*^ haemocytes (Fig. 9D). Altogether, these experiments point to the importance of Eater in binding Gram‐positive bacteria, which is consistent with a previous report 35, but also to a redundant role of NimC1 and Eater in binding Gram‐negative bacteria. Furthermore, they suggest that these two receptors do not play any critical role in bacteria internalization, as *NimC1;eater* mutant showed (rare) engulfment events (Fig. 9B,D arrows).

To further confirm the bacteria adhesion defects, we incubated haemocytes and live fluorescent bacteria either on ice or with Cytochalasin D. Both treatments inhibit the engulfment process, without altering the binding of the bacteria to the phagocytic cell 6. In both conditions (Fig. 9E,F), we observed less bacteria binding to plasmatocytes in the same genotypes that were defective for phagocytosis in our *ex vivo* assays (*eater* ^*1*^ for *S. *aureus, and *NimC1* ^*1*^ *;eater* ^*1*^ for *S. aureus* and *E. coli*, Fig. 8A–D).

### Phagocytosis of latex beads and zymosan yeast particles is impaired in *NimC1* null mutants

**Figure 10:**
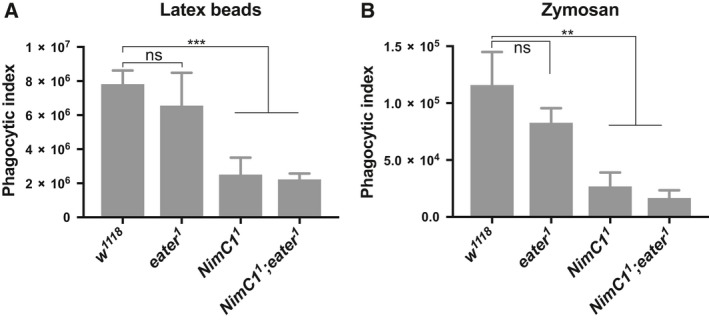
*NimC1* ^*1*^ haemocytes show impaired phagocytosis of latex beads and zymosan particles. (A) Phagocytic index quantification of latex beads (Sigma‐Aldrich, St. Louis, MO, USA) engulfment in haemocytes from *w* ^*1118*^, *eater* ^1^, *NimC1* ^1^ and *NimC1* ^*1*^ *;eater* ^*1*^ L3 wandering larvae after 30 min incubation. Data are represented as mean ± SD from five independent experiments (B) Phagocytic index quantification of AlexaFluor™488 Zymosan (*S. cerevisiae*) BioParticles™ engulfment. Zymosan BioParticles were incubated with *w* ^*1118*^ or mutant haemocytes from L3 wandering larvae for 90 min. Data are represented as mean ± SD from four independent experiments. In (A) and (B), phagocytosis was quantified by flow cytometry, and the fluorescence of extracellular particles quenched by adding trypan blue. **P <* *0.01, ***P <* *0.001, by Student *t* tests. ns, not significant.

To further understand the role of Eater and NimC1 in the phagocytosis process, we proceeded to analyse the uptake of ‘neutral’ latex beads particles. We also tested their role in the phagocytosis of zymosan, a compound found on the cell wall of yeast. While bacteria present at their surface‐specific targets for the engulfing receptors, latex beads can be seen as nonimmunogenic particles, that do not bear any ligands for the phagocyte. We observed that the phagocytic index of latex beads was wild‐type like in *eater* null plasmatocytes. Interestingly, plasmatocytes lacking the NimC1 receptor showed a significantly reduced ability to engulf latex beads, as well as zymosan yeast particles (Fig. 10A,B). Thus, we could uncover a phagocytic defect in the *NimC*1 single mutant only when using particles that do not display bacterial motifs, suggesting that bacteria can bypass NimC1, probably by recruiting other phagocytic receptors, such as Eater.

### Bacteria adhesion and latex beads engulfment are not impaired in *croquemort* and *draper* mutant haemocytes

**Figure 11:**
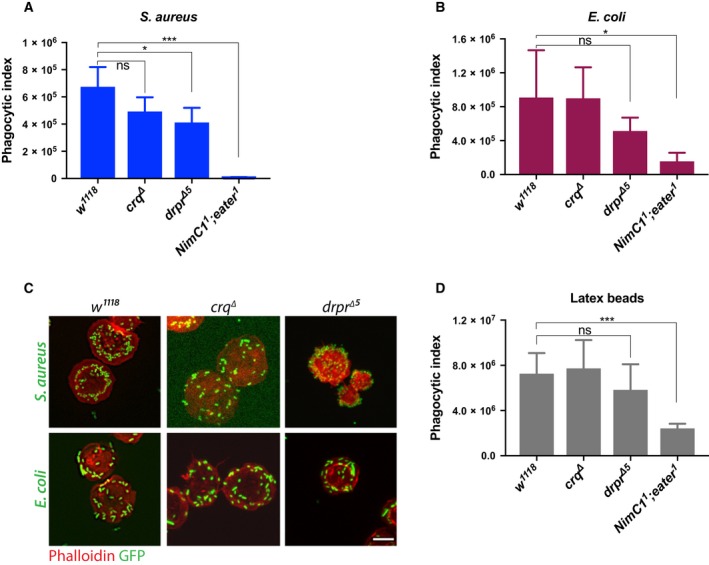
Phagocytosis in *draper* and *croquemort* mutants. Phagocytosis of *Staphylococcus aureus* (A) and *Escherichia coli* (B) AlexaFluor™488 BioParticles™ (Invitrogen) in *crq* ^*∆*^ and *drpr* ^*∆5*^ haemocytes mutants from L3 wandering larvae. *NimC1* ^*1*^ *;eater* ^*1*^ haemocytes were used as negative control. (C) *crq* ^*∆*^ and *drpr* ^*∆5*^ haemocytes mutants show no binding defect of *S. aureus* (upper panel) and *E. coli* (bottom panel) bacteria. Haemocytes from the corresponding genotypes were incubated with live GFP bacteria (green) on ice for 1 h (see Material and Methods section for further details). After fixation with 4% paraformaldehyde, haemocytes were stained with rhodamine phalloidin (red). Scale bar: 10 μm. (D) Phagocytosis of latex beads (Sigma‐Aldrich) in *crq* ^*∆*^ and *drpr* ^*∆5*^ haemocytes mutants from L3 wandering larvae. *NimC1* ^*1*^ *;eater* ^*1*^ haemocytes were used as the negative control. Data are represented as mean ± SD from four independent experiments *P <* *0.05, ***P <* *0.001, by Student *t* tests. ns, not significant.

The drastic effect observed with the *NimC1* ^*1*^ *;eater* ^*1*^ double mutant on phagocytosis and bacteria adhesion led us to explore the contribution of other previously characterized phagocytic receptors using the same assays. Draper and Croquemort are two transmembrane receptors expressed by plasmatocytes, and belong to the Nimrod and CD36 family respectively 14, 36. With SIMU (NimC4), they both play a key role in the engulfment of apoptotic bodies 18, 36, 37, 38, 39. Moreover, a role in *S. aureus* phagocytosis has also been described for Draper and Croquemort, as well as in *E. coli* phagocytosis for Draper 17, 40, 41, 42. Although we did observe a modest decrease in *S. aureus* phagocytosis in *croquemort* and *draper* mutants (called *crq* ^*∆*^ and *drpr* ^*∆5*^ respectively), and *E. coli* in *drpr* ^*∆5*^ (Fig. 11A,B), bacteria adhesion to the haemocytes was not impaired in these mutants (Fig. 11C). This further supports a specific role of Eater as the main tethering receptor in Gram‐positive bacteria phagocytosis. Moreover, *drpr* ^*∆5*^ and *crq* ^*∆*^ haemocytes showed a wild‐type like engulfment of latex beads (Fig. 11D), further indicating a specific role of Eater in microbe uptake, likely via the recognition of a key bacterial surface determinant.

## Discussion and Conclusions

NimC1 was initially identified as an antigen for the plasmatocyte‐specific monoclonal antibody P1. It belongs to the Nimrod gene family that has been implicated in the cellular innate immune response in *Drosophila* 43, 44. A previous study pointed to the importance of NimC1 in the phagocytosis of bacteria, since RNAi‐mediated silencing of this gene resulted in decreased *S. aureus* uptake by plasmatocytes 14. In the present work, we further re‐evaluated the function of the NimC1 protein by using a novel null mutant, revealing its precise role in haemocyte adhesion, proliferation and phagocytic ability.

By performing *ex vivo* spreading assays, we observed that the cell area of adherent *NimC1* null haemocytes was reduced compared to wild‐type control, suggesting that NimC1 works as an adhesion molecule. Consistent with this observation, SEM on spread haemocytes of *NimC1* ^*1*^ mutants revealed a defect in lamellipodia extension. Spreading defects were also observed in *eater* ^*1*^ 16 and *NimC1* ^*1*^ *;eater* ^*1*^ haemocytes. Thus, two structurally related Nimrod receptors, NimC1 and Eater, are involved in lamellipodia extension and haemocyte adhesion. It will be interesting to analyse, in future work, the implications of NimC1 and Eater in haemocyte migration during metamorphosis or wound healing. Our results also indicate that Eater and NimC1 additively regulate haemocyte adherence. In contrast to *eater*‐deficient larvae, NimC1 is, however, not directly required for plasmatocyte sessility *in vivo*. Whether NimC1 contributes to haemocyte sessility through additional scaffold proteins has to be further investigated, even though the present evidence might favour a model where Eater is the only essential protein required for haemocyte sessility 16.

During larval development, the peripheral haemocyte population undergoes a significant proliferation, expanding by self‐renewal 8, 21. Moreover, during these developmental stages, plasmatocytes are characterized by a dynamic behaviour, continuously exchanging between the sessile and circulating state. In 2011, Makhijani *et al*. 21 provided evidence that plasmatocyte proliferation rate is higher in the haematopoietic pockets, where haemocytes cluster on the lateral side of the larval body. At this location, sessile plasmatocytes are in contact with the endings of peripheral neurons, which are thought to provide a trophic environment to the blood cells. More recently, it has been shown that sensory neurons of the peripheral nervous system produce Activin‐β, which turned out to be an important factor in the regulation of haemocyte proliferation and adhesion 45. By analysing the total number of haemocytes in third instar *NimC1* ^*1*^ or *eater* ^*1*^ larvae, we observed that both Eater and NimC1 negatively regulate haemocyte counts in an additive manner. EdU incorporation experiments revealed that the higher haemocyte counts in *NimC1* ^*1*^ *;eater* ^*1*^ mutants were a consequence of an increased haemocyte proliferation rate. It is tempting to speculate that the higher proliferation rate is a secondary consequence of an adhesion defect. Indeed, adherent cells, notably when establishing contacts with other cells, are less proliferative, a process called ‘contact inhibition of proliferation’ 46. Future studies should address how Eater and NimC1 contribute to both adhesion and proliferation, and the direction of causality between these two processes remains to be disentangled. Like plasmatocytes, crystal cells increase in number during larval stages. However, crystal cell proliferation is not due to a self‐renewal mechanism because mature crystal cells do not divide. Instead, a recent study has shown that new crystal cells originate from transdifferentiation of sessile plasmatocytes via a Notch–Serrate‐dependent process 26. In the present study, we show that the *NimC1* deletion does not strongly impact crystal cell formation as both sessile and circulating crystal cell populations were only mildly affected in *NimC1* null larvae. Moreover, NimC1 does not affect the ability to differentiate lamellocytes and to encapsulate parasitoid wasp eggs.

NimC1 was initially identified as a phagocytic receptor, mediating the uptake of *S. aureus* bacteria 14. Contrary to this study, our *ex vivo* phagocytosis assays using the *NimC1* deletion mutant revealed that the uptake of both Gram‐positive and Gram‐negative bacteria was not altered in *NimC1* null haemocytes. We hypothesized that the RNAi approach could have targeted other phagocytic receptors, revealing a stronger phenotype not observed in the single null mutant. Strikingly, phagocytosis of both bacteria types was severely impaired in *NimC1* ^*1*^ *;eater* ^*1*^ haemocytes, suggesting that both receptors contribute synergistically to phagocytosis of both Gram‐negative and Gram‐positive bacteria. At this stage, we cannot exclude that these receptors might indirectly regulate phagocytosis by controlling another receptor directly involved in bacterial recognition, although we judge this hypothesis unlikely. Consistent with our hypothesis, an RNAseq analysis of *eater* deficient versus wild‐type haemocytes did not uncover any role of Eater in the regulation of other phagocytic receptors (data not shown).

Given the marked phagocytosis defect of the *eater* single mutant against *S. aureus*, the contribution of NimC1 was especially noticeable in the case of the Gram‐negative bacterium *E. coli*. Our SEM approach revealed that NimC1 and Eater might contribute together to the early step of bacterial recognition, since *NimC1* ^*1*^ *;eater* ^*1*^ double mutants showed decreased bacterial adhesion. The involvement of NimC1 in *E. coli* binding is consistent with previous *in vitro* work showing that native NimC1 binds bacteria 47. Surprisingly, *NimC1* ^*1*^ and *NimC1* ^*1*^ *;eater* ^*1*^ *,* but not *eater‐*deficient plasmatocytes, showed a significantly reduced ability to engulf latex beads and yeast zymosan particles. A recent study has also revealed a role of NimC1 in the phagocytosis of latex beads using an *in vivo* RNAi approach 48. Thus, Eater and NimC1 have specific properties with regard to phagocytosis. It is interesting to address a parallel with the implication of two Nimrod receptors in bacteria tethering and docking, as shown for apoptotic cells clearance in *Drosophila melanogaster* 18, 49. Tethering receptors usually lack an intracellular domain and are involved in the binding to the dying cell. Docking receptors, instead, are subsequently required to activate intracellular signalling and mediate the internalization and degradation of the particle. In the fruit fly, a good example for tethering and docking receptors are SIMU/NimC4 and Draper respectively 49. A similar dichotomy exists in vertebrates, as Stabilin 2 and TIM‐4 are classified as tethering receptors, whereas the integrins αVβ3 and αVβ5 are grouped as docking/signalling proteins 50, 51. The involvement and cooperation of two receptors of the Nimrod family in bacterial phagocytosis raised the possibility that they might contribute via different mechanisms: binding and internalization. Our current hypothesis is that Eater might work as the main tethering receptor, required for binding to specific motifs present on the bacterial surface. Moreover, given the wild‐type engulfment of latex beads in *eater*, this receptor might be engaged specifically for phagocytosis of microbes. Indeed, the involvement of Eater in bacterial binding was already assessed in previous studies 35, and is consistent with our assays using live fluorescent bacteria and SEM experiments. On the contrary, NimC1 could function in the activation of the subsequent intracellular signalling, maybe as a subunit of a bigger macromolecular complex. We hypothesize that in the presence of cell wall bacterial determinants, such as peptidoglycan, lipopolysaccharide or teichoic acids, microbe phagocytosis can bypass the requirement of NimC1 by providing enough ‘eat me’ signals to Eater. In contrast, the critical role of NimC1 in phagocytosis becomes visible with less immunogenic particles. This would explain why we do not observe any defects in the phagocytosis of *S. aureus* and *E. coli* in *NimC1* single mutant, but only with latex beads (i.e. particles without any bacterial motifs).

Future studies should address how Eater and NimC1 interact, the implication of other possible phagocytic receptors and characterization of their respective ligands. Collectively, our genetic analysis using compound mutants identifies NimC1 and Eater as two critical receptors involved in the initial step of phagocytosis, and notably adhesion to bacteria. While a plethora of receptors have been identified for their role in microbial phagocytosis in *Drosophila*, NimC1 and Eater appear to be the best candidates to directly recognize bacterial ‘eat me’ signals initiating phagocytosis. Our study also provides a valuable tool to better assess the role of phagocytosis during the immune response.

## Materials and methods

### *Drosophila* stocks and methodology

All *Drosophila* stocks were maintained at 25 °C on standard fly medium consisting of 6% cornmeal, 6% yeast, 0.62% agar, 0.1% fruit juice (consisting of 50% grape juice and 50% multifruits+multivitamin juice), supplemented with 10.6 g·L^−1^ moldex and 4.9 mL·L^−1^ propionic acid. Second instar (L2) larvae were selected 48–52 h after egg laying (AEL), middle L3 larvae 72–90 h AEL and third instar (L3) wandering larvae 110–120 h AEL.

Wild‐type *w* ^*1118*^ (BL5905) flies were used as controls, unless indicated otherwise. The following fly lines were used in this study:

**Table:** 

	Details	Source
*w* ^*1118*^	BL5905	Bloomington
*y* ^*1*^ *w* ^*1118*^		
*w* ^*1118*^ *;;eater* ^*1*^		16
*w* ^*1118*^ *;NimC1* ^*1*^		This study
*w* ^*1118*^ *; NimC1* ^*1*^ *;eater* ^*1*^		This study
*w* ^*1118*^ *;;HmlΔdsred.nls*		16
*w* ^*1118*^ *;;eater* ^*1*^ *HmlΔdsred.nls*		This study
*w* ^*1118*^ *;NimC1* ^*1*^ *;HmlΔdsred.nls*		This study
*w* ^*1118*^ *;NimC1* ^*1*^ *;eater* ^*1*^ *HmlΔdsred.nls*		This study
*w* ^*1118*^ *;HmlΔGal4>UAS‐GFP*		52
*w* ^*1118*^ *;HmlΔGal4,UAS‐GFP;eater* ^*1*^		This study
*w* ^*1118*^ *;NimC1* ^*1*^ *,HmlΔGal4,UAS‐GFP*		This study
*w* ^*1118*^ *;NimC1* ^*1*^ *,HmlΔGal4,UAS‐GFP;eater* ^*1*^		This study
*yw,lzGal4>UAS‐GFP*	BL6314	Bloomington
*yw,lzGal4,UAS‐GFP;;eater* ^*1*^		This study
*yw,lzGal4,UAS‐GFP;NimC1* ^*1*^		This study
*yw,lzGal4,UAS‐GFP;NimC1* ^*1*^ *;eater* ^*1*^		This study
*;;draper* ^*Δ5*^		37, 53
*croquemort*		54
*PPO1* ^*Δ*^ *,2* ^*Δ*^ *,3* ^*1*^		55
*UAS‐NimC1‐IR*	VDRC 105799	Vienna *Drosophila* Resource Center
*UAS‐eater‐IR*	VDRC 30097	Vienna *Drosophila* Resource Center
*Relish* ^*E20*^		Described in 34
*;UAS‐NimC1;*		This study

### Gene targeting of *NimC1*

Gene targeting of *NimC1* was performed as follows. The 5′ and 3′ homology arms, of 4.8 kb and 3.7 kb, respectively, were PCR amplified from genomic DNA. The 5′ arm was inserted between *NotI* and *NheI* restriction sites, whereas the 3′ arm was inserted between *SpeI* and *AscI* sites of the gene targeting vector pTV[Cherry]. A donor transgenic stock was generated by transformation of a starting *w1118* (BL5905) stock, and used for hsFLP and hs‐I‐SceI‐mediated gene targeting 19. Using this method, we recorded a 1/2000 knockout efficiency of the F_2_ progeny, that is, 1/2000 offspring were bonafide *NimC1* knockouts.

The following primers were used for PCR genotyping and for testing the functional *NimC1* deletion by RT‐PCR:

**Table:** 

Name	Target gene	Sequence
*eater*_F	*eater*	TAGGAGGTCATAAACGGTCA
*eater*_R	*eater*	CTCAAACGATTTGGACTTTG
*NimC1*_F	*NimC1*	AGTGTGCTCGTTATCTGGAA
*NimC1*_R	*NimC1*	GTTTCCCACTTTCTCGTACC
*NimC1*cDNA_F	*NimC1*	TCGCTTCAAGGACAACTCCC
*NimC*1cDNA_R	*NimC1*	ACACAGTCTCCGAATTGGCA

### Haemocyte counting by flow cytometry

A BD Accuri C6 flow cytometer (Becton Dickinson, San Jose, CA, USA) was used to analyse haemocyte numbers. For each genotype, 15 L2, 5 middle L3 or 5 L3 wandering third instar larvae containing the *Hml∆dsred.nls* marker were bled into 150 μL of Schneider's insect medium (Sigma‐Aldrich) containing 1 nm phenylthiourea (PTU; Sigma‐Aldrich). Before bleeding, larvae were vortexed in PBS 1× for 1 min in order to detach sessile haemocytes 56. About 100 μL of haemocyte suspension was analysed by flow cytometry. Haemocytes were first selected from debris by plotting FSC‐A against SSC‐A on a logarithmic scale in a dot plot. Cells were then gated for singlets by plotting FSC‐H versus FSC‐A. *w* ^*1118*^ and *w* ^*1118*^ *;*;*Hml∆dsred.nls* larvae were used to define the gates for haemocyte population, using a FL2 detector.

### Haemocyte size measurement of free‐floating cells

Third instar (L3) wandering larvae were bled in 1× PBS without calcium and magnesium, supplemented with EDTA 5 mm. Invitrogen™ Tali™ Image‐based Cytometer machine (Carlsbad, CA, USA) was used to measure haemocytes size in suspension of more than 7000 cells per genotype.

### *Ex vivo* larval haemocyte phagocytosis assays

1. *Ex vivo* phagocytosis assay of *E. coli* and *S. aureus* was performed using *E. coli* and *S. aureus* AlexaFluor™488 BioParticles™ (Invitrogen), following manufacturer's instructions. L3 wandering larvae carrying the *Hml∆dsred.nls* haemocytes marker were bled into 150 μL of Schneider's insect medium (Sigma‐Aldrich) containing 1 μm phenylthiourea (PTU; Sigma‐Aldrich). The haemocyte suspension was then transferred to 1.5 mL low binding tubes (Eppendorf, Sigma‐Aldrich) and 2 × 10^7^ AlexaFluor™488 bacteria BioParticles™ were added. The samples were incubated at room temperature for 30 or 60 min to enable phagocytosis, and then placed on ice in order to stop the reaction. The fluorescence of extracellular particles was quenched by adding 0.4% trypan blue (Sigma‐Aldrich) diluted 1/3. Phagocytosis was quantified using a flow cytometer (BD Accuri C6) in order to measure the fraction of cells phagocytosing, and their fluorescent intensity. *w* ^*1118*^ larvae and *Hml∆dsred.nls* larvae with or without bacterial particles were used to define the gates for haemocytes and the thresholds for phagocytosed particle emission. The phagocytic index was calculated as follows:
  $$\text{Fraction of haemocytes phagocytosing}\left(\text{f}\right)=\frac{\left[\text{number of haemocytes in fluorescence positive gate}\right]}{\left[\text{total number of haemocytes}\right]}$$
  Phagocytic index(PI)=[Mean fluorescence intensity of haemocytes in fluorescence positive gate]×f
2. *Ex vivo* phagocytosis assays of *S. marcescens*,* S. epidermidis* and *M. luteus* were performed as follows. Bacterial strains and labelling bacteria with fluorescein isothiocyanate (FITC) are described in 47. The stocks of *S. marcescens* (Szeged Microbial Collection, University of Szeged, Szeged, Hungary; SzMC 0567), *S. epidermidis* (SzMC 14531) and *M. luteus* (SzMC 0264) were used. Bacteria were conjugated by FITC as described by Zsámboki *et al*. Briefly, 10 mL of bacterial culture (OD_600 _= 1.5) was heat inactivated in PBS and the cell pellet was resuspended in 10 mL of 0.25 m carbonate‐bicarbonate buffer pH 9.0. Fluorescein isothiocyanate (FITC) 0.5 mg, dissolved in 100 μL DMSO (Sigma‐Aldrich) was added to the heat‐inactivated bacteria, rotated overnight at 4 °C and washed eight times with PBS. The FITC‐labelled bacteria were resuspended, centrifuged at 11 200 ***g***, the pellet was resuspended to a final concentration of 10%, sodium azide was added as a preservative (0.1%) and the samples were kept at 4 °C until use. Bacteria were washed 5× with PBS prior to the phagocytosis assay. The phagocytic activity of haemocytes was assayed with a protocol similar to 6. Haemocytes were isolated from third instar larvae at room temperature into Shields and Sang M3 insect medium (Sigma‐Aldrich) containing 5% FCS (Gibco, Thermo Fisher, Waltham, MA, USA) supplemented with 1‐phenyl‐2‐thiourea (Sigma‐Aldrich) to prevent melanization. A total of 2–3 × 10.5 haemocytes were incubated with 5–6 × 10^6^ heat‐killed, FITC‐labelled bacteria at room temperature for 40 min in the wells of round bottomed microtitre plates (Gibco) in 100 μL. The fluorescence of extracellular bacteria was quenched by the addition of Trypan blue to the cells in 0.2% final concentration shortly before the actual measurement. The fluorescence intensity of phagocytosed FITC‐labelled bacteria was analysed with a FACS Calibur equipment (BD Accuri C6, Beckton Dickinson). Phagocytic index was calculated as mentioned above.
3. Phagocytosis of green fluorescent 1 μm latex beads (Sigma‐Aldrich) and AlexaFluor™488 Zymosan BioParticles™ (Invitrogen) was performed following the same procedure described in 1), with the exception that haemocytes without the *Hml∆dsred.nls* marker were used. 1 × 10^5^ Zymosan BioParticles™ and 0.2 μg of latex beads were added to each sample.

Given the difference in haemocyte numbers per larva between the genotypes, we bled 6 *w* ^*1118*^ (*BL5905*) larvae, 4 *eater* ^*1*^ and *NimC1* ^*1*^ larvae and 3 *NimC1* ^*1*^ *;eater* ^*1*^ larvae.

### Proliferation assays

Cell proliferation was assessed by 5‐ethynyl‐2′‐deoxyuridine (EdU) labelling. Second instar, or middle L3 larvae were fed at 29 °C with 1 mm 5‐ethynyl‐2 deoxyuridine (EdU) in fly food for 4 h. Larvae were bled individually in 30 μL Schneider medium (Gibco) containing 1 nm phenylthiourea (PTU; Sigma‐Aldrich). Haemocytes were allowed to settle for 30 min before being fixed in 4% paraformaldehyde PBS. Click‐iT™ EdU Imaging Kit (Invitrogen) was used to stain *Hml∆dsred.nls* haemocyte populations. Cells were finally stained with 1/15 000 dilution of 4′,6‐ diamidino‐2‐phenylindole DAPI (Sigma‐Aldrich) and mounted in antifading agent Citifluor AF1 (Citifluor Ltd., Hatfield, PA, USA). The proliferation rate was determined by counting the number of EdU‐positive cells over the whole *Hml∆dsred.nls* haemocyte population. At least six animals were analysed per genotype.

### Scanning electron microscopy

Samples for SEM were prepared as follows. Six wandering third instar larvae were bled into 50 μL of Schneider's insect medium (Sigma‐Aldrich) containing 1 μm phenylthiourea (PTU; Sigma‐Aldrich). The collected haemolymph was incubated on a glass coverslip for 20 min for spreading assay, or 30 min with bacteria for phagocytosis assay, before being fixed for 1 h with 1.25% glutaraldehyde in 0.1 m phosphate buffer, pH 7.4. Samples were then washed in cacodylate buffer (0.1 m, pH 7.4), fixed again in 0.2% osmium tetroxide in the same washing buffer and then dehydrated in graded alcohol series. Samples underwent critical point drying and Au/Pd coating (4 nm). Scanning electron micrographs were taken with a field emission scanning electron microscope Merlin, Zeiss NTS, Oerzen, Embsen, Germany.

### Transmission electron microscopy

Third instar wandering larvae were bled in 50 μL of Schneider's insect medium (Sigma‐Aldrich) containing 1 μm phenylthiourea (PTU; Sigma‐Aldrich). The collected haemolymph was incubated with bacteria on a glass coverslip for 1 h before being fixed for 2 h with 2% paraformaldehyde + 2.5% glutaraldehyde in 0.1 m phosphate buffer, pH 7.4. Samples were then washed in cacodylate buffer (0.1 m, pH 7.4), fixed again in 1% osmium tetroxide and potassium ferrocyanide 1.5% in cacodylate buffer. After washes in distilled water, samples were stained in 1% uranyl acetate in water, washed again, and then dehydrated in graded alcohol series (2× 50%, 1× 70%, 1× 90%, 1× 95%, 2× 100%). Embedding was performed first in 1 : 1 Hard EPON and ethanol 100%, and afterwards in pure EPON, before being embedded on coated glass slides and placed at 60 °C overnight. Images were acquired with a FEI Tecnai Spirit 120 kV(FEI Company, Eagle, The Netherlands).

### Binding assay with live fluorescent bacteria

#### Cytochalasin D treatment

L3 wandering larvae were bled into 120 μL of Schneider's insect medium (Sigma‐Aldrich) containing 1 μm phenylthiourea (PTU; Sigma‐Aldrich). Haemocytes were allowed to adhere on the glass slide for 1 h before being treated for another 60 min with 1 μm of Cytochalasin D. After drug treatment, haemocytes were incubated directly on the slide with live fluorescent *S. aureus* or *E. coli* bacteria always in the presence of Cytochalasin D for 60 min. After fixation in 4% paraformaldehyde PBS, rhodamine phalloidin staining (Molecular Probes™, Eugene, OR, USA) was performed. Finally, cells were stained with 1/15 000 dilution of 4′,6‐diamidino‐2‐phenylindole DAPI (Sigma‐Aldrich) and mounted in antifading agent Citifluor AF1 (Citifluor Ltd.).

#### Phagocytosis inhibition by cold temperature

L3 wandering larvae were bled into cold Schneider's insect medium (Sigma‐Aldrich) containing 1 μm phenylthiourea (PTU; Sigma‐Aldrich) on a previously chilled glass slide. After larva bleeding, haemocytes and bacteria were incubated directly on the prechilled slide, in cold Schneider's medium, on ice for 60 min. Fixation and staining procedures were performed as described above.

#### Fluorescent bacteria

The *E. coli* GFP strain was obtained by transforming *E. coli* K12 with a synthetic sfGFP coding sequence cloned in a pBAD backbone (Gibco, ThermoFisher) by Gibson assembly. sfGFP induction was obtained by growing the bacteria in LB + 0.1% arabinose overnight prior to the binding assay. The *S. aureus* GFP strain is described in Ref. 57.

### Haemocyte phalloidin staining and cell area measurement

Five wandering third instar larvae were bled on a microscope slide into 120 μL of 1× PBS containing 1 μm phenylthiourea (PTU; Sigma‐Aldrich). The haemocytes were then allowed to adhere for 30 min, before being fixed in 4% paraformaldehyde PBS. Phalloidin staining was performed with diluted 1/100 AlexaFluor488‐ or rhodamine phalloidin (Molecular Probes™). Finally, cells were stained with a 1/15 000 dilution of 4′,6‐ diamidino‐2‐phenylindole DAPI (Sigma‐Aldrich) and mounted in anti‐fading agent Citifluor AF1 (Citifluor Ltd.). Samples were imaged with an Axioplot Imager.Z1 Zeiss (Oberkochen, Germany) coupled to an AxioCam MRm camera (Zeiss).

For cell area measurements, haemocytes were captured with a 20× objective on GFP, RFP and DAPI channels. Individual images were then loaded into a CellProfiler pipeline (http://www.cellprofiler.org). In order to define the cell area, cell nuclei were first detected using data from the DAPI channel. Cell limits were then defined by expanding the nuclei signal to the edges of the GFP channel. Cell areas were computed from this segmentation analysis, and cell area of 750 cells of each genotype was quantified.

### Haemocytes visualization through larva cross sectioning

Third instar larvae of the indicated genotypes were fixed in 4% paraformaldehyde PBS for 48 h at 4 °C. Afterwards, larvae were embedded using OCT medium in a Tissue‐Tek cryomolds (Sakura, Alphen aan den Rijn, The Netherlands). Transverse sections of 4–5‐μm thickness were cut using Leica CM1959 cryostat. Finally, sections were fixed again for 15 min in 4% paraformaldehyde PBS, prior to rhodamine phalloidin (Molecular Probes™) staining. Samples were imaged with an Axioplot Imager.Z1 Zeiss coupled to an AxioCam MRm camera (Zeiss).

### Crystal cell counting methods

At least 10 third instar larvae were heated in 1 mL of phosphate‐buffered saline (PBS) at 67 °C for 20 min in Eppendorf tubes. For quantification analysis, black puncta were counted in the posterior‐most segments A6, A7 and A8. Pictures were taken with a Leica DFC300FX camera (Leica Microsystems AG, Heerbrugg, Switzerland) and Leica Application Suite right after heating.

For quantification of crystal cells by flow cytometry, we crossed wild‐type or mutant *lzGal4>UAS‐GFP* flies with the corresponding *HmlΔdsred.nls w* ^*1118*^ or mutant flies. Larvae form the resulting offspring were used to determine the number of crystal cells (*lzGal4>UAS‐GFP*) and the ratio of crystal cells among the total haemocyte population (*lzGal4>UAS‐GFP* / *HmlΔdsred.nls*). Four larvae of each genotype were bled into 150 μL 1× PBS containing 1 μm phenylthiourea (PTU; Sigma‐Aldrich) and 0.1% paraformaldehyde to block crystal cell rupture. Seventy‐five microlitres of the haemocyte suspension was analysed by flow cytometry. Haemocytes were first selected from debris by plotting FSC‐A against SSC‐A on a logarithmic scale in a dot plot. Cells were then gated for singlets by plotting FSC‐H versus FSC‐A. FL1 and FL2 detectors were used for *lzGal4>UAS‐GFP* and *Hml∆dsred.nls* events respectively.

### Wounding experiment

Wandering third instar larvae were pricked dorsally near the posterior end of the animal, using a sterile needle (diameter ~ 5 μm). Pictures of melanised larvae were taken 20 min after pricking, with a Leica DFC300FX camera and Leica Application Suite.

### Wasp infestation and quantification of fly survival to *Leptopilina boulardi* infestation

For wasp infestation experiments, 30 synchronized second instar (L2) larvae were placed on a pea‐sized mound of fly food within a custom‐built wasp trap in the presence of three female *L. boulardi* (strain NS1c, described in Ref. 58) for 2 h. Quantification of fly survival was performed as follows. Parasitized larvae were kept at room temperature and scored daily for flies or wasps emergence. The number of eclosed flies and wasps was subtracted of the initial number of exposed larvae and set as dead larvae/pupae. Pictures of melanised eggs were taken with a Leica DFC300FX camera and Leica Application Suite.

### Infection experiments and qRT‐PCR

Systemic infections (septic injuries) were performed by pricking third instar larvae dorsally near the posterior end of the animal using a thin needle previously dipped into a concentrated pellet (OD_600_ ~ 200) of bacteria. After septic injury, larvae were incubated at 29 °C. After 4 h, the animals were collected, and total RNA extraction was performed using TRIzol reagent (Invitrogen). RNA quality and quantity were determined using a NanoDrop ND‐1000 spectrophotometer (NanoDrop Technologies, Inc., Wilmington, DE, USA) and 500 ng of total RNA was used to generate cDNA using SuperScript II (Invitrogen). Quantitative PCR was performed on cDNA samples using the LightCycler 480 SYBR Green Master Mix (Roche, Basel, Switzerland). Expression values were normalized to *RpL32*.

### Statistical analysis

Experiments were repeated at least three times independently and values are represented as the mean ± standard deviation (SD). Data were analysed using graphpad prism 7.0 (San Diego, CA, USA). *P*‐values were determined with Mann–Whitney tests, unless indicated otherwise. For phagocytic index measurement experiments, data successfully passed a Shapiro–Wilk normality test (α = 0.05, *n* = 9), so that we could assume that samples follow Gaussian distribution. Therefore, significance tests were performed using Students *t* test.

## Conflict of interest

The authors declare no conflict of interest.

## Author contributions

CM and BL conceived and designed the project. CM, AJB and ER contributed to the generation of the *NimC1* ^*1*^ mutant and other tools used in this study. JD and IA performed the wasp experiments, and EK and IA did the phagocytosis assay with *S. epidermidis*,* M. luteus* and *Se. marcescens*. CM performed all the other experiments of the study. CM and BL wrote the paper.

## References

[1] Oczypok EA , Oury TD & Chu CT (2013) It's a cell‐eat‐cell world: autophagy and phagocytosis. Am J Pathol 182, 612–622. 23369575 10.1016/j.ajpath.2012.12.017 PMC3589073

[2] Aderem A (2003) Phagocytosis and the inflammatory response. J Infect Dis 187, S340–S345. 12792849 10.1086/374747

[3] Arandjelovic S & Ravichandran KS (2015) Phagocytosis of apoptotic cells in homeostasis. Nat Immunol 16, 907–917. 26287597 10.1038/ni.3253 PMC4826466

[4] Rosales C & Uribe‐Querol E (2017) Phagocytosis: a fundamental process in immunity. Biomed Res Int 2017, 9042851. 28691037 10.1155/2017/9042851 PMC5485277

[5] Stuart LM & Ezekowitz RAB (2005) Phagocytosis: elegant complexity. Immunity 22, 539–550. 15894272 10.1016/j.immuni.2005.05.002

[6] Pearson AM , Baksa K , Rämet M , Protas M , McKee M , Brown D & Ezekowitz RAB (2003) Identification of cytoskeletal regulatory proteins required for efficient phagocytosis in *Drosophila* . Microbes Infect 5, 815–824. 12919849 10.1016/s1286-4579(03)00157-6

[7] Agaisse H , Burrack LS , Philips J , Rubin EJ , Perrimon N & Higgins DE (2005) Genome‐wide RNAi screen for host factors required for intracellular bacterial infection. Science 1248, 1116008. 10.1126/science.1116008 16020693

[8] Lanot R , Zachary D , Holder F & Meister M (2001) Postembryonic hematopoiesis in *Drosophila* . Dev Biol 230, 243–257. 11161576 10.1006/dbio.2000.0123

[9] Tepass U , Fessler LI , Aziz A & Hartenstein V (1994) Embryonic origin of hemocytes and their relationship to cell death in *Drosophila* . Development 120, 1829–1837. 7924990 10.1242/dev.120.7.1829

[10] Gold KS & Brückner K (2015) Macrophages and cellular immunity in *Drosophila melanogaster* . Semin Immunol 27, 357–368. 27117654 10.1016/j.smim.2016.03.010 PMC5012540

[11] Stuart LM & Ezekowitz RA (2008) Phagocytosis and comparative innate immunity: learning on the fly. Nat Rev Immunol 8, 131–141. 18219310 10.1038/nri2240

[12] Ulvila J , Vanha‐Aho LM & Rämet M (2011) *Drosophila* phagocytosis – still many unknowns under the surface. APMIS 119, 651–662. 21917002 10.1111/j.1600-0463.2011.02792.x

[13] Kocks C , Cho JH , Nehme N , Ulvila J , Pearson AM , Meister M , Strom C , Conto SL , Hetru C , Stuart LM *et al* (2005) Eater, a transmembrane protein mediating phagocytosis of bacterial pathogens in *Drosophila* . Cell 123, 335–346. 16239149 10.1016/j.cell.2005.08.034

[14] Kurucz É , Márkus R , Zsámboki J , Folkl‐Medzihradszky K , Darula Z , Vilmos P , Udvardy A , Krausz I , Lukacsovich T , Gateff E *et al* (2007) Nimrod, a putative phagocytosis receptor with EGF repeats in *Drosophila* plasmatocytes. Curr Biol 17, 649–654. 17363253 10.1016/j.cub.2007.02.041

[15] Somogyi K , Sipos B , Pénzes Z , Kurucz É , Zsámboki J , Hultmark D & Andó I (2008) Evolution of genes and repeats in the Nimrod superfamily. Mol Biol Evol 25, 2337–2347. 18703524 10.1093/molbev/msn180

[16] Bretscher AJ , Honti V , Binggeli O , Burri O , Poidevin M , Kurucz E , Zsamboki J , Ando I & Lemaitre B (2015) The Nimrod transmembrane receptor Eater is required for hemocyte attachment to the sessile compartment in *Drosophila melanogaster* . Biol Open 4, 355–363. 25681394 10.1242/bio.201410595 PMC4359741

[17] Hashimoto Y , Tabuchi Y , Sakurai K , Kutsuna M , Kurokawa K , Awasaki T , Sekimizu K , Nakanishi Y & Shiratsuchi A (2009) Identification of lipoteichoic acid as a ligand for Draper in the phagocytosis of *Staphylococcus aureus* by *Drosophila* hemocytes. J Immunol 183, 7451–7460. 19890048 10.4049/jimmunol.0901032

[18] Kurant E , Axelrod S , Leaman D & Gaul U (2008) Six‐microns‐under acts upstream of Draper in the glial phagocytosis of apoptotic neurons. Cell 133, 498–509. 18455990 10.1016/j.cell.2008.02.052 PMC2730188

[19] Baena‐Lopez LA , Alexandre C , Mitchell A , Pasakarnis L & Vincent J‐P (2013) Accelerated homologous recombination and subsequent genome modification in *Drosophila* . Development 140, 4818–4825. 24154526 10.1242/dev.100933 PMC3833436

[20] Small JV , Stradal T , Vignal E & Rottner K (2002) The lamellipodium: where motility begins. Trends Cell Biol 12, 112–120. 11859023 10.1016/s0962-8924(01)02237-1

[21] Makhijani K , Alexander B , Tanaka T , Rulifson E & Bruckner K (2011) The peripheral nervous system supports blood cell homing and survival in the *Drosophila* larva. Development 138, 5379–5391. 22071105 10.1242/dev.067322 PMC3222213

[22] Makhijani K & Brückner K (2012) Of blood cells and the nervous system: hematopoiesis in the *Drosophila* larva. Fly (Austin) 6, 254–260. 23022764 10.4161/fly.22267 PMC3519660

[23] Markus R , Laurinyecz B , Kurucz E , Honti V , Bajusz I , Sipos B , Somogyi K , Kronhamn J , Hultmark D & Ando I (2009) Sessile hemocytes as a hematopoietic compartment in *Drosophila melanogaster* . Proc Natl Acad Sci USA 106, 4805–4809. 19261847 10.1073/pnas.0801766106 PMC2660760

[24] Zettervall C‐J , Anderl I , Williams MJ , Palmer R , Kurucz E , Ando I & Hultmark D (2004) A directed screen for genes involved in *Drosophila* blood cell activation. Proc Natl Acad Sci 101, 14192–14197. 15381778 10.1073/pnas.0403789101 PMC521135

[25] Honti V , Csordás G , Márkus R , Kurucz É , Jankovics F & Andó I (2010) Cell lineage tracing reveals the plasticity of the hemocyte lineages and of the hematopoietic compartments in *Drosophila melanogaster* . Mol Immunol 47, 1997–2004. 20483458 10.1016/j.molimm.2010.04.017

[26] Leitão AB & Sucena É (2015) *Drosophila* sessile hemocyte clusters are true hematopoietic tissues that regulate larval blood cell differentiation. Elife 2015, 1–38. 10.7554/eLife.06166 PMC4357286 25650737

[27] Holz A (2003) The two origins of hemocytes in *Drosophila* . Development 130, 4955–4962. 12930778 10.1242/dev.00702

[28] Krzemien J , Oyallon J , Crozatier M & Vincent A (2010) Hematopoietic progenitors and hemocyte lineages in the *Drosophila* lymph gland. Dev Biol 346, 310–319. 20707995 10.1016/j.ydbio.2010.08.003

[29] Jung S‐H (2005) The *Drosophila* lymph gland as a developmental model of hematopoiesis. Development 132, 2521–2533. 15857916 10.1242/dev.01837

[30] Louradour I , Sharma A , Morin‐Poulard I , Letourneau M , Vincent A , Crozatier M & Vanzo N (2017) Reactive oxygen species‐dependent Toll/NF‐κB activation in the *Drosophila* hematopoietic niche confers resistance to wasp parasitism. Elife 6, 1–22. 10.7554/eLife.25496 PMC5681226 29091025

[31] Vlisidou I & Wood W (2015) *Drosophila* blood cells and their role in immune responses. FEBS J 282, 1368–1382. 25688716 10.1111/febs.13235

[32] Stofanko M , Kwon SY & Badenhorst P (2010) Lineage tracing of lamellocytes demonstrates *Drosophila* macrophage plasticity. PLoS One 5, e14051. 21124962 10.1371/journal.pone.0014051 PMC2988793

[33] Anderl I , Vesala L , Ihalainen TO , Vanha‐aho LM , Andó I , Rämet M & Hultmark D (2016) Transdifferentiation and proliferation in two distinct hemocyte lineages in *Drosophila melanogaster* larvae after Wasp infection. PLoS Pathog 12, 1–34. 10.1371/journal.ppat.1005746 PMC4945071 27414410

[34] Neyen C , Bretscher AJ , Binggeli O & Lemaitre B (2014) Methods to study *Drosophila* immunity. Methods 68, 116–128. 24631888 10.1016/j.ymeth.2014.02.023

[35] Chung YSA & Kocks C (2011) Recognition of pathogenic microbes by the *Drosophila* phagocytic pattern recognition receptor eater. J Biol Chem 286, 26524–26532. 21613218 10.1074/jbc.M110.214007 PMC3143617

[36] Franc NC , Dimarcq JL , Lagueux M , Hoffmann J & Ezekowitz RAB (1996) Croquemort, a novel drosophila hemocyte/macrophage receptor that recognizes apoptotic cells. Immunity 4, 431–443. 8630729 10.1016/s1074-7613(00)80410-0

[37] Freeman MR , Delrow J , Kim J , Johnson E & Doe CQ (2003) Unwrapping glial biology: Gcm target genes regulating glial development, diversification, and function. Neuron 38, 567–580. 12765609 10.1016/s0896-6273(03)00289-7

[38] Manaka J , Kuraishi T , Shiratsuchi A , Nakai Y , Higashida H , Henson P & Nakanishi Y (2004) Draper‐mediated and phosphatidylserine‐independent phagocytosis of apoptotic cells by *Drosophila* hemocytes/macrophages. J Biol Chem 279, 48466–48476. 15342648 10.1074/jbc.M408597200

[39] Franc NC , Heitzler P , Ezekowitz RA & White K (1999) Requirement for croquemort in phagocytosis of apoptotic cells in *Drosophila* . Science 284, 1991–1994. 10373118 10.1126/science.284.5422.1991

[40] Shiratsuchi A , Mori T , Sakurai K , Nagaosa K , Sekimizu K , Lee BL & Nakanishi Y (2012) Independent recognition of *Staphylococcus aureus* by two receptors for phagocytosis in *Drosophila* . J Biol Chem 287, 21663–21672. 22547074 10.1074/jbc.M111.333807 PMC3381130

[41] Stuart LM , Deng J , Silver JM , Takahashi K , Tseng AA , Hennessy EJ , Ezekowitz RAB & Moore KJ (2005) Response to *Staphylococcus aureus* requires CD36‐mediated phagocytosis triggered by the COOH‐terminal cytoplasmic domain. J Cell Biol 170, 477–485. 16061696 10.1083/jcb.200501113 PMC2171464

[42] Cuttell L , Vaughan A , Silva E , Escaron CJ , Lavine M , Van Goethem E , Eid JP , Quirin M & Franc NC (2008) Undertaker, a *Drosophila* Junctophilin, links Draper‐mediated phagocytosis and calcium homeostasis. Cell 135, 524–534. 18984163 10.1016/j.cell.2008.08.033

[43] Somogyi K , Sipos B , Pénzes Z & Andó I (2010) A conserved gene cluster as a putative functional unit in insect innate immunity. FEBS Lett 584, 4375–4378. 20951134 10.1016/j.febslet.2010.10.014

[44] Cinege G , Zsámboki J , Vidal‐Quadras M , Uv A , Csordás G , Honti V , Gábor E , Hegedűs Z , Varga GIB , Kovács AL *et al* (2017) Genes encoding cuticular proteins are components of the Nimrod gene cluster in *Drosophila* . Insect Biochem Mol Biol 87, 45–54. 28633893 10.1016/j.ibmb.2017.06.006

[45] Makhijani K , Alexander B , Rao D , Petraki S , Herboso L , Kukar K , Batool I , Wachner S , Gold KS , Wong C *et al* (2017) Regulation of *Drosophila* hematopoietic sites by Activin‐β from active sensory neurons. Nat Commun 8, 15990. 28748922 10.1038/ncomms15990 PMC5537569

[46] Fagotto F & Gumbiner BM (1996) Cell contact‐dependent signaling. Dev Biol 180, 445–454. 8954717 10.1006/dbio.1996.0318

[47] Zsámboki J , Csordás G , Honti V , Pintér L , Bajusz I , Galgóczy L , Andó I & Kurucz É (2013) Drosophila Nimrod proteins bind bacteria. Cent Eur J Biol 8, 633–645.

[48] Hao Y , Yu S , Luo F & Jin LH (2018) Jumu is required for circulating hemocyte differentiation and phagocytosis in *Drosophila* . Cell Commun Signal 16, 95. 30518379 10.1186/s12964-018-0305-3 PMC6280549

[49] Shklover J , Levy‐Adam F & Kurant E (2015) Apoptotic Cell Clearance in Development, 1st edn Elsevier Inc., Amsterdam. 10.1016/bs.ctdb.2015.07.024 26431572

[50] Kobayashi N , Karisola P , Peña‐Cruz V , Dorfman DM , Jinushi M , Umetsu SE , Butte MJ , Nagumo H , Chernova I , Zhu B *et al* (2007) TIM‐1 and TIM‐4 glycoproteins bind phosphatidylserine and mediate uptake of apoptotic cells. Immunity 27, 927–940. 18082433 10.1016/j.immuni.2007.11.011 PMC2757006

[51] Park SY , Jung MY , Kim HJ , Lee SJ , Kim SY , Lee BH , Kwon TH , Park RW & Kim IS (2008) Rapid cell corpse clearance by stabilin‐2, a membrane phosphatidylserine receptor. Cell Death Differ 15, 192–201. 17962816 10.1038/sj.cdd.4402242

[52] Sinenko SA & Mathey‐Prevot B (2004) Increased expression of *Drosophila* tetraspanin, Tsp68C, suppresses the abnormal proliferation of ytr‐deficient and Ras/Raf‐activated hemocytes. Oncogene 23, 9120–9128. 15480416 10.1038/sj.onc.1208156

[53] MacDonald JM , Beach MG , Porpiglia E , Sheehan AE , Watts RJ & Freeman MR (2006) The *Drosophila* cell corpse engulfment receptor Draper mediates glial clearance of severed axons. Neuron 50, 869–881. 16772169 10.1016/j.neuron.2006.04.028

[54] Han C , Song Y , Xiao H , Wang D , Franc NC , Yeh Jan L & Jan YN (2014) Epidermal cells are the primary phagocytes in the fragmentation and clearance of degenerating dendrites in *Drosophila* . Neuron 81, 544–560. 24412417 10.1016/j.neuron.2013.11.021 PMC3995171

[55] Dudzic JP , Kondo S , Ueda R , Bergman CM & Lemaitre B (2015) *Drosophila* innate immunity: regional and functional specialization of prophenoloxidases. BMC Biol 13, 1–16. 26437768 10.1186/s12915-015-0193-6 PMC4595066

[56] Petraki S , Alexander B & Brückner K (2015) Assaying Blood Cell Populations of the Drosophila melanogaster Larva. J Vis Exp, 105, 52733. 10.3791/52733 PMC4692709 26650404

[57] Needham AJ , Kibart M , Crossley H , Ingham PW & Foster SJ (2004) *Drosophila melanogaster* as a model host for *Staphylococcus aureus* infection. Microbiology 150, 2347–2355. 15256576 10.1099/mic.0.27116-0

[58] Varaldi J , Fouillet P , Ravallec M , López‐Ferber M , Boulétreau M & Fleury F (2003) Infectious behavior in a parasitoid. Science 302, 1930. 14564013 10.1126/science.1088798

[59] Honti V , Cinege G , Csordás G , Kurucz É , Zsámboki J , Evans CJ , Banerjee U & Andó I (2013) Variation of NimC1 expression in *Drosophila* stocks and transgenic strains. Fly (Austin) 7, 263–266. 23899817 10.4161/fly.25654 PMC3896499

[60] Rizki TM , Rizki RM & Grell EH (1980) A mutant affecting the crystal cells in *Drosophila melanogaster* . Wilhelm Roux's Arch Dev Biol 188, 91–99. 10.1007/BF00848799 28304971

[61] Kurucz E , Zettervall C , Sinka R , Vilmos P , Pivarcsi A , Ekengren S , Hegedüs Z , Ando I & Hultmark D (2003) Hemese, a hemocyte‐specific transmembrane protein, affects the cellular immune response in *Drosophila* . Proc Natl Acad Sci USA 100, 2622–2627. 12598653 10.1073/pnas.0436940100 PMC151390

